# Severe vegetation degradation associated with different disturbance types in a poorly managed urban recreation destination in Iran

**DOI:** 10.1038/s41598-021-99261-5

**Published:** 2021-10-04

**Authors:** Mohammad Bagher Erfanian, Juha M. Alatalo, Hamid Ejtehadi

**Affiliations:** 1 Quantitative Plant Ecology and Biodiversity Research Lab., Department of Biology, Faculty of Science, Ferdowsi University of Mashhad, PO BOX 9177948974, Mashhad, Iran; 2 Environmental Science Center, Qatar University, Doha, Qatar

**Keywords:** Restoration ecology, Urban ecology

## Abstract

Recreational activities worldwide have major impacts on the environment. This study examined the impact of different kinds of recreational activities on plant communities in a highly visited park in Mashhad, Iran. Vegetation in the park was sampled along 41 random 10-m transects with different human disturbances (trails, dirt roads, campsites) and undisturbed communities. Life form spectrum, species composition, species and phylogenetic diversity were determined for all communities. Disturbance increased the frequency of therophytes, but decreased the frequency of chamaephytes and percentage vegetated area. Recreational-mediated disturbance had variable impact on species composition, but decreased species and phylogenetic diversity compared with undisturbed areas. Roads and campsites caused the greatest damage, while trails had the smallest negative impact on vegetation. This study showed that damage to (semi-)natural park vegetation differs with recreation activity. This finding can help prioritise management activities to minimise negative impacts of recreation activities on local vegetation. The current visitor load to the urban park studied here appears too high to be sustainable over time, so better monitoring and restrictions on visitor numbers may be needed to minimise the negative impacts on park vegetation. The camping impacts can be managed by creating clusters of designated campsites to spatially concentrate the impact area. Low-impact practices should be communicated to visitors.

## Introduction

Natural areas used as outdoor recreation destinations suffer various adverse environmental impacts from visitor activities^1,2^. Among outdoor activities, hiking and camping are popular worldwide^3^. There is a large body of literature describing the adverse effects of these two activities on natural vegetation and soil in different regions^4–6^. Frequently reported negative impacts of hiking and camping on the vegetation in recreation destinations include changes in species composition, reduced biodiversity, shorter vegetation, more patches of bare soil and complete removal of vegetation^2,4,5,7–10^. Recreation activities are a major source of disturbance in arid regions, and their impacts on arid ecosystems are poorly understood^11–13^. There is a geographical bias in recreation ecology studies, with few published reports from developing countries^6,14^. Moreover, impacts of recreation vary greatly as a response to disturbance intensity and vegetation type^15^. Managers in developing countries have to refer to studies in other parts of the world with a different evolutionary history of natural ecosystems and thus the results can be misleading for these managers, especially in preparing a proper conservation programme^2,14^. An appropriate management strategy should preserve recreation values to enhance positive social impacts^16^.

Iran occupies an area of around 1.64 million km^2^ and has a diverse flora^17^. Approximately 30% of 8000 recorded plant species in Iran are endemic and some habitats in the country are located in the Irano-Anatolian and Caucasus global biodiversity hotspots^18^. Land use changes and grazing are the major threats to the habitats of Iran^18–22^. These disturbances, together with lack of knowledge^23^, make it difficult to plan appropriate management programmes for Iran’s natural habitats.

Mashhad, located in north eastern Iran, is the second-largest and most-visited city in the country. It is also among the most visited religious cities in the world^19^. Recreation destinations in Mashhad are visited by large numbers of visitors (residents and tourists), especially during the holiday period. These destinations are located in the Khorassan-Kopet Dagh floristic province, which falls within the Irano-Anatolian biodiversity hotspot and is home to many range-restricted endemic species. Only around 8% of the Khorassan-Kopet Dagh is officially protected, while remaining parts are generally unregulated^21^. In a study on an environmentally friendly managed ski-piste located 70 km from Mashhad, Erfanian et al.^24^ concluded that appropriate management of this recreation destination could help restore the natural vegetation in areas with a long history of heavy grazing. To our knowledge, there have been no other published studies about recreation destinations in Mashhad and nearby areas. The aim of the present study was to fill this research gap.

The area selected for the study was a mountainous urban park (Khorshid Park) in Mashhad where recreation activities are not regulated. As a result, its vegetation has been affected by multiple recreational activities for a decade. The hypothesis tested was that different recreation activities shape plant communities in the study area and that recurrent disturbances lead to severe vegetation disturbance. The specific objective was to quantify the disturbances caused by each recreational activity carried out in the study area (hiking, camping, driving on dirt roads). The investigations performed were necessary because characterising the environmental impacts of these disturbances is the first step in devising a suitable management programme.

## Materials and methods

### Study area

The 400-ha Khorshid Park in the west of Mashhad city (36.2971°N, 59.4828°E; 1100–1500 m a.s.l.) is a popular urban recreation destination. The park was established in 2007 to provide a recreation destination for trekkers, mountaineers and campers^25^. It is located on the north-facing slope of the Binalood Mountains, a major mountain range within the Khorassan-Kopet Dagh floristic province^21^. The area is characterized by a Mediterranean xeric oceanic bioclimate^26^. Khorshid Park is the access route to Zou peak, the highest peak in the Mashhad. Dirt and asphalt roads have been constructed within the park, to allow access to campsites and trails. The most intensively used area within the park is a camping zone in the lower part of the park. Camping occurs along the roads. Khorshid Park is one of the main recreation destinations for residents and for pilgrims to Mashhad. This park hosts around 10,000 daily visitors in general and over 75,000 daily visitors during the holidays^25^. Different recreation activities of these visitors induce disturbances that affect the vegetation in the park. At the time of the study, the park was managed solely for recreational purposes, and not to ensure the conservation of flora and fauna in the area.

The natural vegetation in the park mainly consists of shrubs, perennial and annual grasses and forbs, with no natural tree species present. There are no data available on the total number of species present in Khorshid Park, but a floristic study on an adjacent valley of similar area (~ 450 ha) identified 311 vascular plant species^27^. Planting of trees is ongoing in Khorshid Park, but mainly with *Pinus brutia* var. *eldarica* and *Robinia pseudoacacia*, which are not native to the area. Grazing by cattle is prohibited in this urban park and there are no indigenous large herbivores in the area.

### Field study

The study area had homogenous vegetation before the park was established and there is no evident environmental factor in the area that could lead to vegetation heterogeneity. The natural vegetation of the area was sampled along 10-m transects, in which there were no tree species. Considering the nature of the disturbances occurring in the park (i.e. trails, dirt roads, campsites), a line-intercept sampling method was used^28^. Disturbed plant communities were identified as those located within 1 m of the disturbance source. Random transects, running in parallel with the disturbance sources, were sampled at 30 cm distance from trails, dirt roads (hereafter ‘roads’) and campsites. To compare the disturbed communities with (semi-)natural communities, control transects lying well away from any disturbance source were used. These control transects were randomly sampled in areas with no sign of any disturbances. A total of 41 transects (10 control, 10 trail, 10 road and 11 campsites) were studied. No transects were located under the canopy of the planted trees. Along each transect, all species of vascular plants and vegetation cover were recorded. The life form of each species was identified based on the Raunkiær classification system^28^.

### Data analysis

#### Physiognomy and vegetated area

To visualise the impacts of disturbances on the physiognomy of plant communities, the Raunkiær life form spectrum of each community was plotted using the ggplot2 package^29^ in R (version 3.5)^30^. It has been reported that different disturbances can change the life form spectrum of a plant community^28^. Here, the median of the total vegetation cover of the control transects was used as a base, and percentage change in vegetation cover of other groups relative to that value was calculated. Box plots comparing these values were drawn in R. One-way analysis of variance (ANOVA) was used to compare the total vegetation cover of the groups. Finally, Duncan's multiple range test was used for multiple post hoc comparisons. ANOVA was performed in the base R and Duncan’s test in the agricolae package^31^.

#### Species composition

Transformation-based principal component analysis (tb-PCA) was used to visualise the species composition of each group. Prior to this analysis, the species composition data were Hellinger-transformed to eliminate the effects of double-zeros^32^. PCA was then applied on the transformed data, using functions in the vegan package in R^33^.

#### Biodiversity

The species diversity of the communities studies was compared based on the Hill numbers^34^. Species richness (q = 0 in Hill numbers), the exponential of the Shannon–Wiener index (q = 1 in Hill numbers), and the reciprocal of the Gini-Simpson index (q = 2 in Hill numbers) were then calculated. These indices evaluate community species diversity at the level of rare, frequent and dominant species, respectively^19,34^. The coverage-based rarefaction and extrapolation method was used to eliminate the effects of unequal sampling effort (e.g. unequal number of samples) on the comparisons^35^. The species diversity of the communities was estimated at the same coverage level. Moreover, for statistical inferences, a 95% confidence interval (CI) for each index was calculated, using a 999-time bootstrapping approach in the iNEXT package^36^. The results were reported as the percentage change in species diversity in the disturbed communities relative to the species diversity of the control community.

An angiosperm phylogeny of all species collected in the transects was used, and a phylogenetic tree was created using the V.Phylomaker package^37^. Non-angiosperms (i.e. *Ephedra*) were excluded, to prevent addition of long branches that would create outliers in the phylogenetic diversity calculations^38^. Phylogenetic diversity measures use phylogenetic differences between species, with communities with higher phylogenetic value given priority in conservation^39^. Hill’s phylogenetic diversity indices were used to compare phylogenetic diversity among the communities and categorise it into one of three levels: rare (q = 0), frequent (q = 1), and dominant (q = 2) species. Coverage-based rarefaction and extrapolation methods were used for this and 95% CIs were calculated for each index, using the iNEXT-PD package^36,40,41^. The phylogenetic diversity results were reported as percentage change relative to the control community, using the same approach as applied for species diversity.

## Results

### Life form and vegetation cover

**Figure 1:**
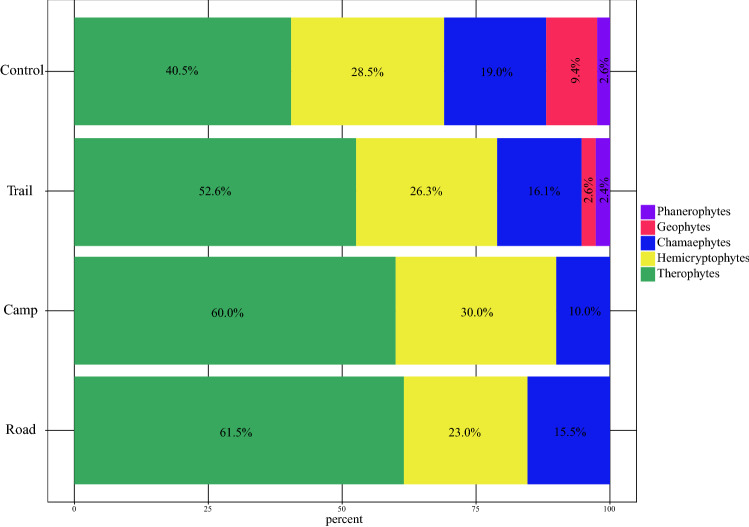
Raunkiær life form spectrum for the three disturbance types (hiking trail, dirt road, campsite) and control communities.

Therophytes were found to be the dominant life form in the transects. Comparing the communities, all disturbed communities had higher amounts of therophytes than the control, but reduced percentage cover of chamaephytes (e.g. shrubs). Depending on the type of disturbance, hemicryptophytes increased or decreased in abundance compared with the control community. Phanerophytes and geophytes were not found in the campsite and road communities. The life form spectrum for each community is presented in Fig. 1. A list of all species recorded in this study is presented in Supplementary Material S1.

**Figure 2:**
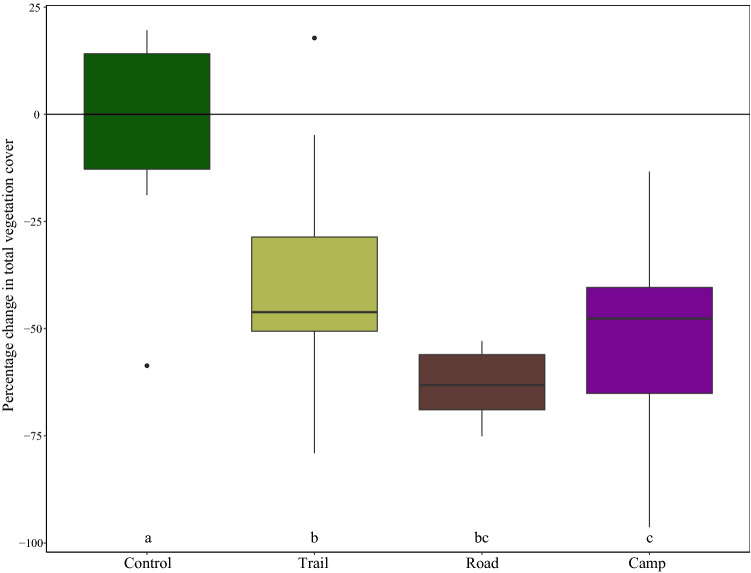
Box plot showing the variation in total vegetation cover in the disturbed (hiking trail, dirt road, campsite) and control communities. Letters indicate the results of post hoc test, with different letters indicating statistically significant differences between the categories.

All three disturbed communities showed a decrease in vegetation cover (Fig. 2), with a reduction compared with the control of 48%, 60%, and 50% for trail, road, and campsite, respectively. Therefore, campsite and road plant communities showed the lowest amount of vegetated area. The ANOVA and Duncan’s test results revealed that this reduction was significant for all disturbed communities compared with the control. The results of Shapiro–Wilk normality test and Levene’s test for homogeneity of variance in vegetation cover of the four communities are provided in Supplementary Material S2.

### Species composition

**Figure 3:**
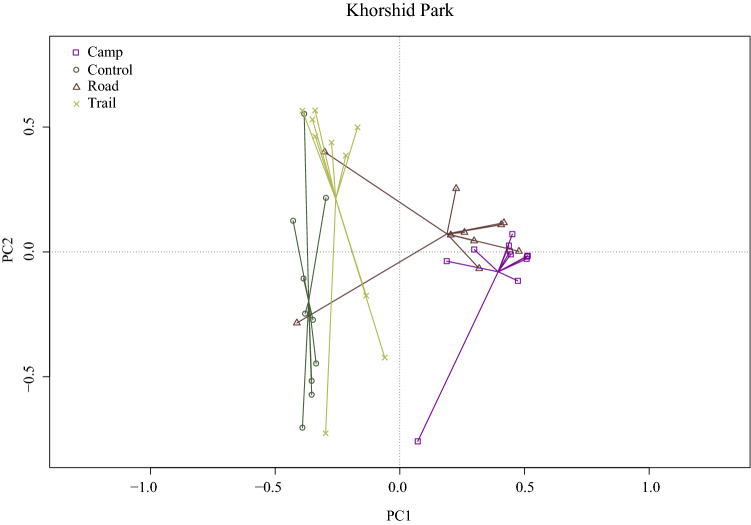
Transformation-based principal component analysis (tb-PCA) plot of variation in species composition along transects sampled in Khorshid Park. Communities (hiking trail, dirt road, campsite, control) are indicated with different colours, each symbol represents a transect.

The results of tb-PCA are shown in Fig. 3. Moderate species composition dissimilarity was observed between the plant communities in Khorshid Park. Campsite and road communities exhibited a greater species composition change compared with the control community. Trail communities remained relatively similar to the control, while campsite and road communities were similar to each other.

### Biodiversity

#### Species diversity

**Figure 4:**
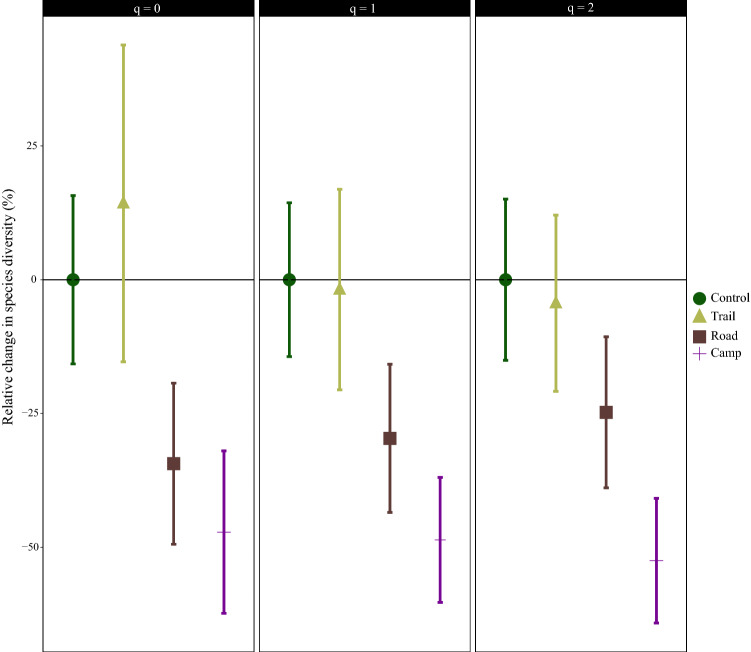
Relative change in species diversity (species richness (q = 0), the exponential of the Shannon–Wiener index (q = 1) and the reciprocal of the Gini-Simpson index (q = 2)) in different plant communities along transects in Khorshid Park compared with the control community value. The error bars are 95% confidence intervals, obtained from a 999 bootstrapping procedure.

The measured base coverage level for the study area, on which all species and phylogenetic diversity calculations were based, was 0.90. The campsite and road communities had significantly (alpha = 0.05) lower species richness (q = 0; diversity at the level of rare species) than the control and trail communities (Fig. 4). A total reduction of 47% and 34% in species richness was observed for the campsite and road communities, respectively. Trail communities showed a non-significant (14%) increase in species richness (Fig. 4). At the level of frequent species (q = 1) or the exponential of the Shannon diversity, all communities showed decreased diversity compared with the controls. The campsite community showed a 48% reduction in diversity and had significantly lower species diversity than the control and trail communities. However, there was no significant difference in species diversity of the control, trail and road communities at the level of frequent species (Fig. 4). Considering the species diversity at the level of dominant species (q = 2) or the reciprocal of the Gini-Simpson index, all communities again showed reduced species diversity compared with the control. A 52% reduction was found for the campsite community, which had significantly lower species diversity than the other communities at the level of dominant species (Fig. 4).

#### Phylogenetic diversity

**Figure 5:**
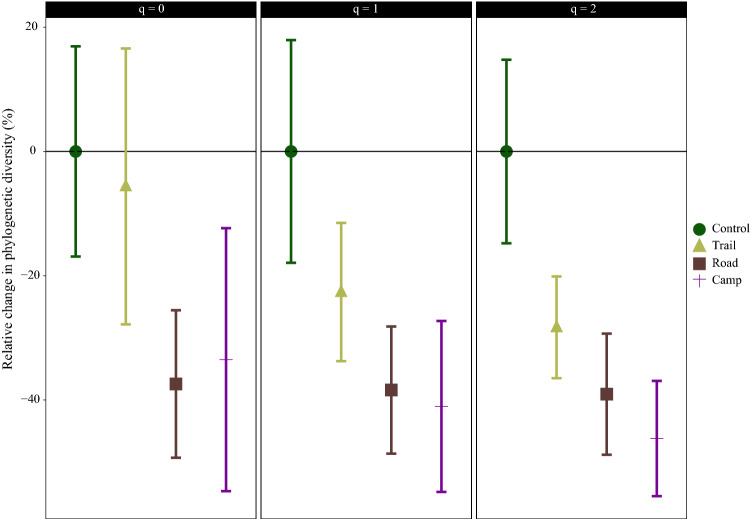
Relative change in phylogenetic diversity in different plant communities along transects in the Khorshid Park compared with the control community value. The phylogenetic diversity at the level of rare (q = 0), frequent (q = 1) and dominant (q = 2) plants are presented. The error bars are 95% confidence intervals, obtained from a 999 bootstrapping procedure.

All disturbed communities showed decreased phylogenetic richness (q = 0) compared with the control community (Fig. 5). However, only the road community, for which a 37% reduction in phylogenetic richness was observed, had significantly lower phylogenetic richness (q = 0) than the control. For phylogenetic diversity at the level of frequent species (q = 1), all three disturbed communities showed a decrease and the road and campsite communities had significantly lower phylogenetic diversity than the control community. The reduction observed was 38% and 41% for the road and campsite communities, respectively. There was no significant difference between the control and trail communities. Considering the phylogenetic diversity at the level of dominant species (q = 2), all three disturbed communities had significantly lower phylogenetic diversity than the control community. The campsite community also had significantly lower phylogenetic diversity than the trail community. The reduction in phylogenetic diversity was 28%, 39% and 46% for the trail, road and campsite communities, respectively.

## Discussion

Comparison of plant communities around three different types of disturbance (roads, trails, campsites) in the heavily used urban recreation destination of Khorshid Park revealed that all these disturbances affected the (semi-)natural vegetation. All communities showed an increased percentage of therophytes compared with the control community. Increased therophyte incidence is reported to be a reliable indicator of vegetation disturbance^19^. According to Monz et al.^5^, changes in the life form spectrum of plant communities are a sensitive indicator of resource changes due to recreation-mediated disturbances. Among the three types of disturbance studied here, the campsite vegetation was the most severely damaged, and plant communities near hiking trails experienced the least damage.

### Effects of trails on plant communities

Plant communities near hiking trails showed a significant reduction in the phylogenetic diversity of dominant species and total vegetation cover compared with the control community. A reduction in vegetation cover has been identified previously as a critical effect of trails on vegetation in multiple studies in different regions^7,14,42–46^. The increased incidence of therophytes and the decreases in chamaephytes and hemicryptophytes in the trail community might be due to a shift from stress-tolerant plants to annual plants. To our knowledge, phylogenetic diversity changes due to recreation activities have not been assessed in previous studies. In the present study, the results showed that trails caused a non-significant phylogenetic diversity reduction in nearby plant communities. A non-significant decrease in species diversity of heath vegetation near trails in alpine Sweden has been reported previously^47^. It has been suggested that trails can change the species composition of nearby vegetation^48^, but our results revealed that trail communities in Khorshid Park had similar species composition to the control community.

### Effects of roads on plant communities

In addition to trails, the impacts of dirt roads in the park were assessed, because different types of track can have different impacts on plant communities^49^. Compared with the trails, plant communities located near roads showed worse status. Wilkerson and Whitman^50^ also reported that tracks used by vehicles showed higher disturbance than pedestrian trails. Road communities had the highest percentage of therophytes in the present study and, along with campsite plant communities, had the lowest total vegetation cover, which is an indicator of severe vegetation fragmentation. This is a frequent negative effect of recreational activities^42^. Comparing the species and phylogenetic diversity of the road and control communities revealed that, except for the reciprocal of the Gini-Simpson index (q = 2), there was a significant decrease in the road community (Fig. 4). A moderate difference in species composition was observed between the control and road plant communities. This is mainly because roadside plant communities experience different environmental conditions (e.g. soil moisture) than the natural plant communities in the area^51^. Newly introduced species that are generally not part of the natural community are frequently found growing in roadside communities^52,53^.

### Effects of campsites on plant communities

The greatest decrease in total vegetation cover was observed for the campsite plant community, due to high trampling intensity around the campsites. Increased percentage of bare soil due to camping activities has been reported previously for different regions worldwide^4,15,54–56^. Monz et al.^5^ reported complete removal of plant species cover as an outcome of camping in the Northern Forest, USA. The lowest percentage cover of chamaephytes (e.g. shrubs) was recorded for the campsite community, which could be an indicator of shrub use for building campfires. A highly significant biodiversity decrease was detected when comparing the campsite and control communities, except for phylogenetic richness (q = 0; Fig. 5). The plant community at the campsites differed from the control vegetation. Species composition change caused by intensive camping has been reported previously^4^. Plant communities near roads and campsites were similar, possibly because in Khorshid Park roads are mainly used to access the campsites. This shows the role of roads in transporting species propagules to different areas, as previously reported for multiple regions^52,57^. It should be noted that vegetation composition is more sensitive to recreation disturbances than vegetation cover, also restoring natural vegetation has a slow rate^4^.

### Management implications

Sustainable use of the park should maintain fundamental ecological processes in the area. Khorshid Park and similar areas are necessary for people to learn about nature and spend time in it, and management needs to balance high-quality visitor experience with minimising associated impacts on vegetation. Our analysis showed that roads and campsites induce the highest disturbances in the park, so regulations should immediately concentrate on these two activities. The camping impact can be managed by creating car campgrounds or clusters of designated campsites. These could be placed in areas that are more sustainable, which would help spatially concentrate the area of camping impact. A camping containment strategy could be used in the park. Low-impact practices (i.e. ‘Leave No Trace’) should be communicated to visitors. Khorshid Park need to be maintained for recreational values and environmental values, to ensure best experiences for people and least damage to the vegetation. Long-term monitoring of the park is needed to follow up on the results.

### Limitations

Due to quarantine caused by COVID-19, Khorshid Park campsites were closed during the present study. Therefore, the change in vegetation cover brought about by this type of visitor-related disturbance might be even greater than recorded in this study. Another limitation was that, due to continuous disturbance, we were unable to sample the plant communities along asphalt roads in the park. Finally, although invasion by non-native species is a crucial component of recreation-mediated disturbance, we were unable to evaluate this owing to lack of reliable data on non-native species in Iran.

## Conclusions

This study is the first to examine the impacts of recreational disturbances on plant communities in a part of Khorassan-Kopet Dagh floristic province, in the Irano-Anatolian biodiversity hotspot. The results showed that different recreation-related disturbances (hiking trails, campsites, dirt roads) have negative impacts on the (semi-)natural vegetation in the area. The results also revealed the importance of considering phylogenetic diversity when assessing the impacts of recreation activities on plant communities. The main impacts identified here should be targeted in future monitoring and protection programmes for Khorshid Park. Other recreation destinations in Mashhad city and throughout Iran should also be monitored and managed.

## Supplementary Information

Supplementary Information.

## Data Availability

Data available from the corresponding author on reasonable request.
