# Supplementary material for: Severe vegetation degradation associated with different disturbance types in a poorly managed urban recreation destination in Iran
Source: Sci Rep. 2021 Oct 4;11:19695. doi: 10.1038/s41598-021-99261-5 (PMC8490473; doi:10.1038/s41598-021-99261-5)
Supplement: Supplementary file 1 — Supplementary Information. [file 41598_2021_99261_MOESM1_ESM.docx]

S1. List of species recorded in Khorshid park during the study

| Species | Transect type |
| --- | --- |
| *Acantholimon pterostegium* | Control - Trail |
| *Acanthophyllum sordidum* | Control |
| *Acinos graveolens* | Road |
| *Aegilops tauschii* | Road - Trail |
| *Aegilops triuncialis* | Control - Trail - Road - Camp |
| *Allium xiphopetalum* | Control |
| *Alyssum desertorum* | Control - Road - Trail |
| *Andrachne telephioides* | Road |
| *Arenaria serpyllifolia* | Control - Trail |
| *Artemisia deserti* | Control - Trail - Road - Camp |
| *Artemisia scoparia* | Trail |
| *Astragalus verus* | Control |
| *Boissiera squarrosa* | Control - Trail |
| *Bromus danthoniaea* | Control - Trail - Road |
| *Bromus tectorum* | Control - Trail - Road |
| *Callipeltis cucullaris* | Control - Trail |
| *Capparis spinosa* | Trail |
| *Carex pachystylis* | Control - Trail - Road - Camp |
| *Centaurea virgata* | Control - Trail |
| *Chenopodium album* | Road - Camp |
| *Cousinia congesta* | Control - Trail - Road |
| *Crepis sancta* | Trail - Camp |
| *Cynodon dactylon* | Road - Camp |
| *Delphinium semibarbatum* | Control |
| *Diarthron vesiculosum* | Control - Trail - Road - Camp |
| *Echinops orientalis* | Trail |
| *Ephedra intermedia* | Control |
| *Erodium sp* | Trail |
| *Eryngium bungei* | Control |
| *Euphorbia szovitsii* | Camp |
| *Euphorbia spinidens* | Control |
| *Helianthemum salicifolium* | Control |
| *Heliotropium europaeum* | Road - Camp |
| *Heterocaryum subsessile* | Trail |
| *Heteropappus altaicus* | Control - Trail |
| *Hordeum murinum* | Trail - Road - Camp |
| *Lactuca orientalis* | Control - Trail - Road |
| *Lactuca seriola* | Control - Trail - Road - Camp |
| *Lallemantia royleana* | Trail |
| *Malcolmia africana* | Control - Trail - Camp |
| *Malva neglecta* | Camp |
| *Melica persica* | Trail |
| *Minuartia meyeri* | Control - Trail - Road |
| *Noaea mucronata* | Control - Trail |
| *Onosma dichroantha* | Trail |
| *Poa bulblosa* | Control - Trail - Road - Camp |
| *Polygonum sp* | Control - Road - Camp |
| *Pulicaria gnaphalodes* | Road |
| *Reseda lutea* | Control - Trail - Road - Camp |
| *Rosa persica* | Control - Road - Camp |
| *Scabiosa rotata* | Control - Trail - Road - Camp |
| *Stipa sp* | Control - Trail |
| *Taeniatherum caputmedusae* | Control - Trail - Road - Camp |
| *Taraxacum sp* | Control |
| *Thalictrum isopyroides* | Control |
| *Thalictrum sultanabadense* | Control |
| *Trichodesma incanum* | Control |
| *Tulipa biflora* | Control - Trail |
| *Tulipa biflora* | Trail |
| *Verbascum songaricum* | Control |
| *Vulpia persica* | Control - Trail - Road - Camp |
| *Ziziphora tenuior* | Control - Trail |

S2. Shapiro-Wilk normality test and Levene's test values for homogeneity of variance in vegetation cover of the four plant community types studied

| The Shapiro-Wilk normality test | |
| --- | --- |
| Trail | W = 0.94387, p-value = 0.5968 |
| Road | W = 0.9356, p-value = 0.5052 |
| Control | W = 0.85939, p-value = 0.07505 |
| Camp | W = 0.98269, p-value = 0.9792 |
| Levene's Test for Homogeneity of Variance | |
| F value = 1.5223 Pr(>F) = 0.2248 | |
